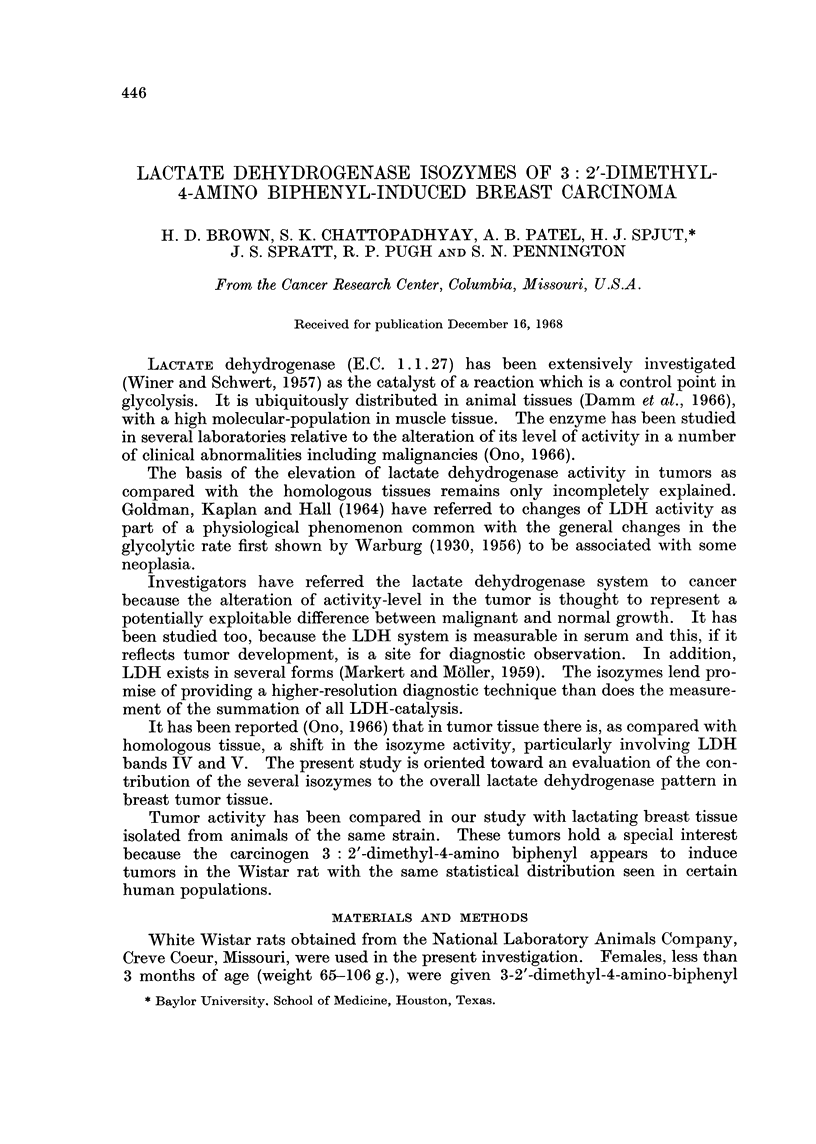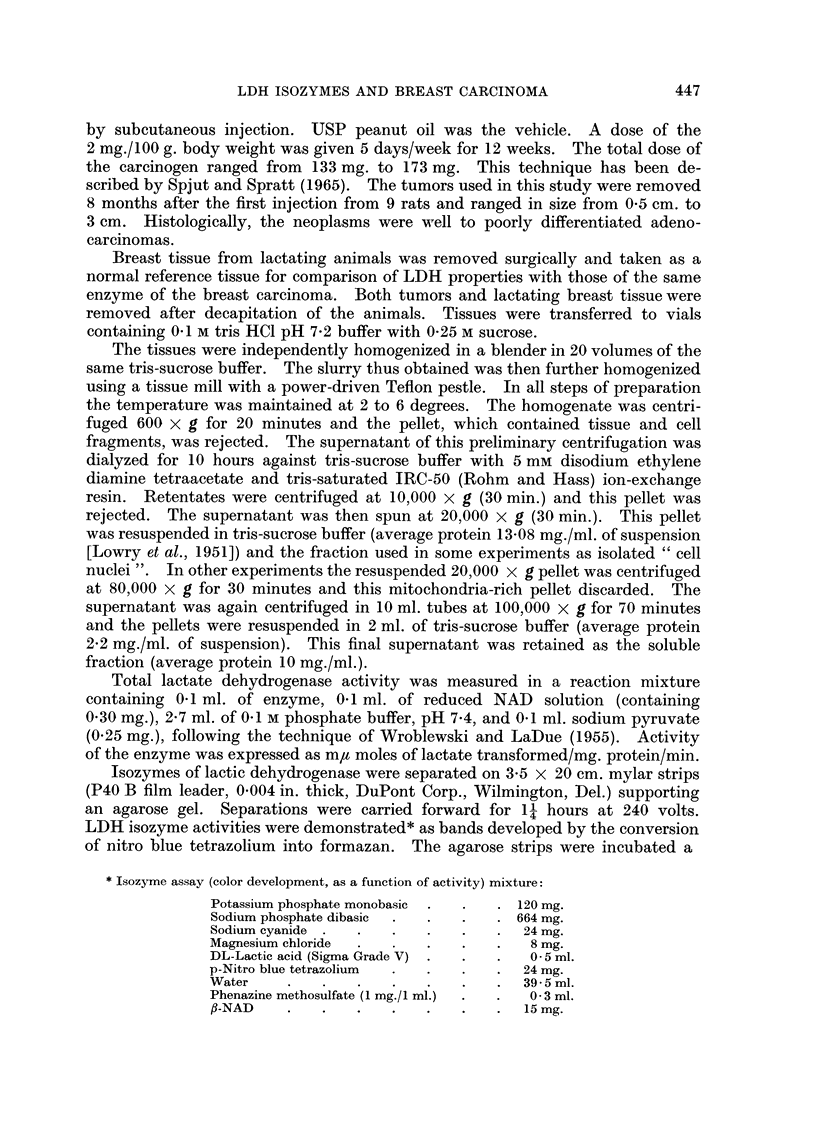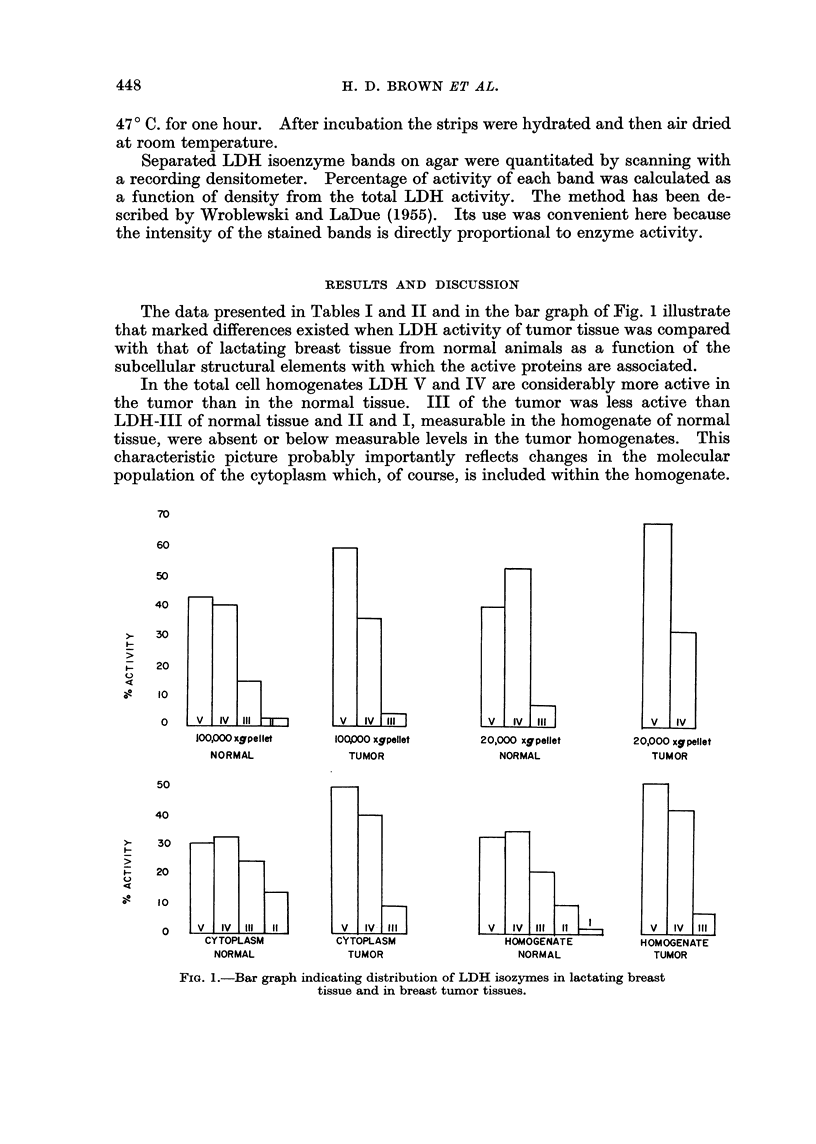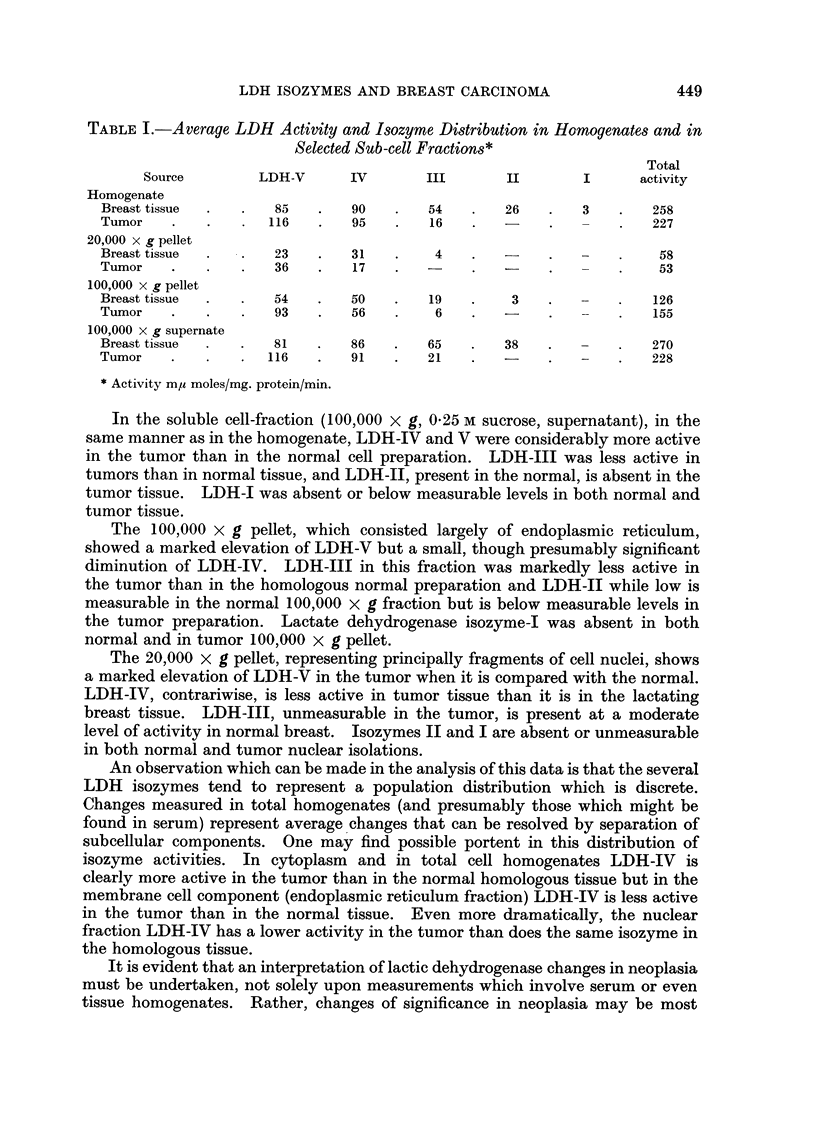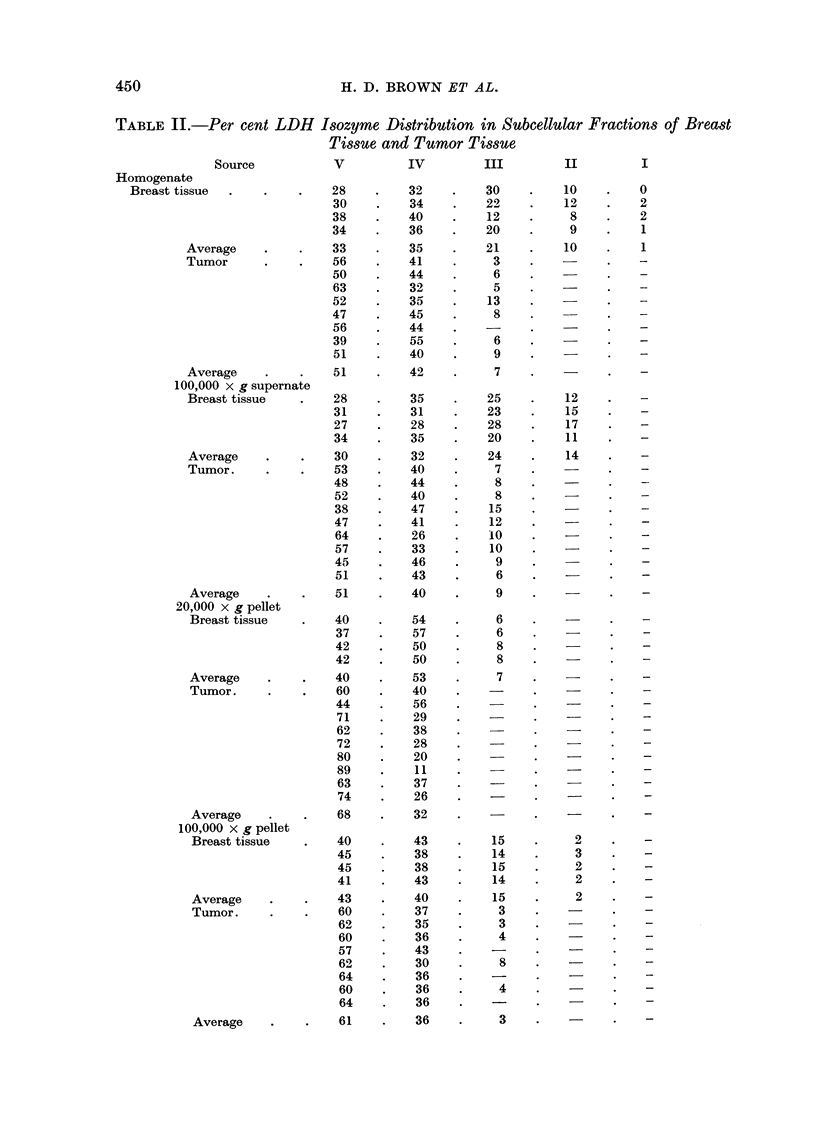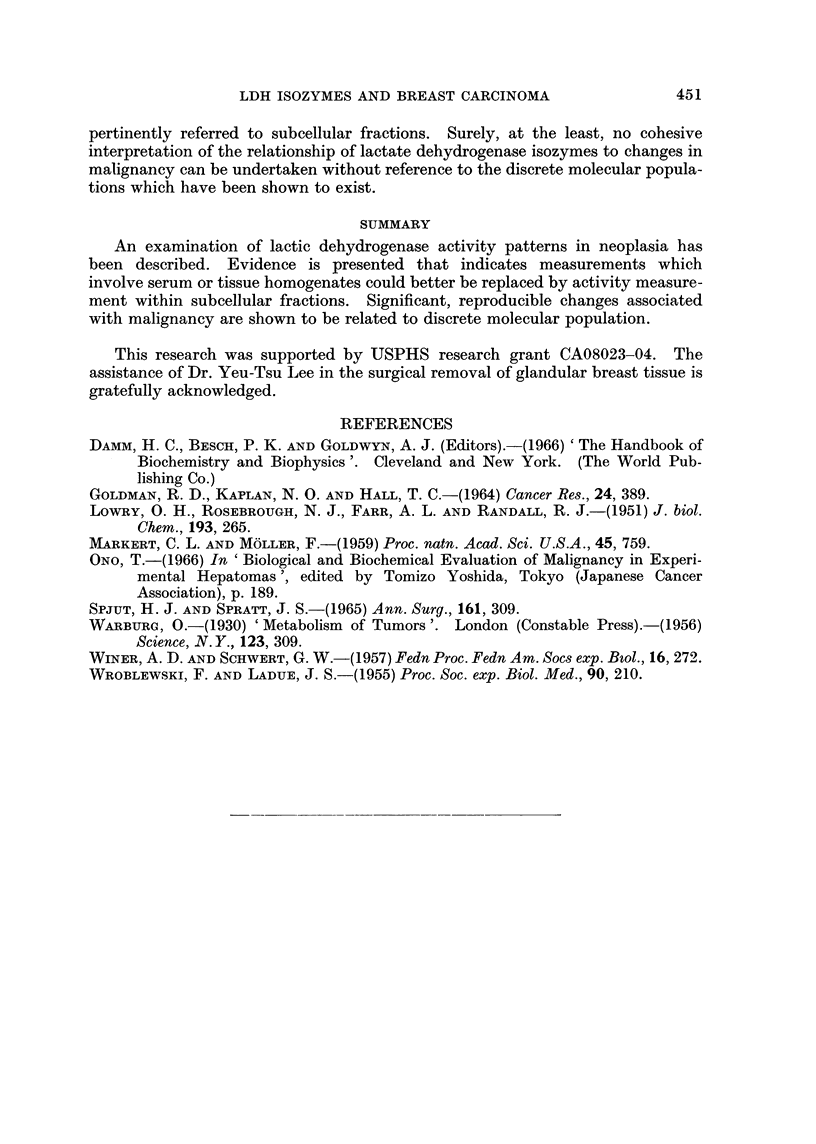# Lactate dehydrogenase isozymes of 3-2'-dimethyl-4-amino biphenyl-induced breast carcinoma.

**DOI:** 10.1038/bjc.1969.57

**Published:** 1969-06

**Authors:** H. D. Brown, S. K. Chattopadhyay, A. B. Patel, H. J. Spjut, J. S. Spratt, R. P. Pugh, S. N. Pennington


### LACTATE DEHYDROGENASE ISOZYMES OF 3 : 2′-DIMETHYL-4-AMINO BIPHENYL-INDUCED BREAST CARCINOMA

H. D. BROWN, S. K. CHATTOPADHYAY, A. B. PATEL, H. J. SPJUT,*
J. S. SPRATT, R. P. PUGH AND S. N. PENNINGTON

*From the Cancer Research Center, Columbia, Missouri, U.S.A.*

Received for publication December 16, 1968

LACTATE dehydrogenase (E.C. 1.1.27) has been extensively investigated (Winer and Schwert, 1957) as the catalyst of a reaction which is a control point in glycolysis. It is ubiquitously distributed in animal tissues (Damm *et al.*, 1966), with a high molecular-population in muscle tissue. The enzyme has been studied in several laboratories relative to the alteration of its level of activity in a number of clinical abnormalities including malignancies (Ono, 1966).

The basis of the elevation of lactate dehydrogenase activity in tumors as compared with the homologous tissues remains only incompletely explained. Goldman, Kaplan and Hall (1964) have referred to changes of LDH activity as part of a physiological phenomenon common with the general changes in the glycolytic rate first shown by Warburg (1930, 1956) to be associated with some neoplasia.

Investigators have referred the lactate dehydrogenase system to cancer because the alteration of activity-level in the tumor is thought to represent a potentially exploitable difference between malignant and normal growth. It has been studied too, because the LDH system is measurable in serum and this, if it reflects tumor development, is a site for diagnostic observation. In addition, LDH exists in several forms (Markert and Möller, 1959). The isozymes lend promise of providing a higher-resolution diagnostic technique than does the measurement of the summation of all LDH-catalysis.

It has been reported (Ono, 1966) that in tumor tissue there is, as compared with homologous tissue, a shift in the isozyme activity, particularly involving LDH bands IV and V. The present study is oriented toward an evaluation of the contribution of the several isozymes to the overall lactate dehydrogenase pattern in breast tumor tissue.

Tumor activity has been compared in our study with lactating breast tissue isolated from animals of the same strain. These tumors hold a special interest because the carcinogen 3 : 2′-dimethyl-4-amino biphenyl appears to induce tumors in the Wistar rat with the same statistical distribution seen in certain human populations.

### MATERIALS AND METHODS

White Wistar rats obtained from the National Laboratory Animals Company, Creve Coeur, Missouri, were used in the present investigation. Females, less than 3 months of age (weight 65–106 g.), were given 3-2′-dimethyl-4-amino-biphenyl

* Baylor University, School of Medicine, Houston, Texas.

by subcutaneous injection. USP peanut oil was the vehicle. A dose of the 2 mg./100 g. body weight was given 5 days/week for 12 weeks. The total dose of the carcinogen ranged from 133 mg. to 173 mg. This technique has been described by Spjut and Spratt (1965). The tumors used in this study were removed 8 months after the first injection from 9 rats and ranged in size from 0·5 cm. to 3 cm. Histologically, the neoplasms were well to poorly differentiated adenocarcinomas.

Breast tissue from lactating animals was removed surgically and taken as a normal reference tissue for comparison of LDH properties with those of the same enzyme of the breast carcinoma. Both tumors and lactating breast tissue were removed after decapitation of the animals. Tissues were transferred to vials containing 0·1 M tris HCl pH 7·2 buffer with 0·25 M sucrose.

The tissues were independently homogenized in a blender in 20 volumes of the same tris-sucrose buffer. The slurry thus obtained was then further homogenized using a tissue mill with a power-driven Teflon pestle. In all steps of preparation the temperature was maintained at 2 to 6 degrees. The homogenate was centrifuged 600 × *g* for 20 minutes and the pellet, which contained tissue and cell fragments, was rejected. The supernatant of this preliminary centrifugation was dialyzed for 10 hours against tris-sucrose buffer with 5 mM disodium ethylene diamine tetraacetate and tris-saturated IRC-50 (Rohm and Hass) ion-exchange resin. Retentates were centrifuged at 10,000 × *g* (30 min.) and this pellet was rejected. The supernatant was then spun at 20,000 × *g* (30 min.). This pellet was resuspended in tris-sucrose buffer (average protein 13·08 mg./ml. of suspension [Lowry *et al.*, 1951]) and the fraction used in some experiments as isolated " cell nuclei ". In other experiments the resuspended 20,000 × *g* pellet was centrifuged at 80,000 × *g* for 30 minutes and this mitochondria-rich pellet discarded. The supernatant was again centrifuged in 10 ml. tubes at 100,000 × *g* for 70 minutes and the pellets were resuspended in 2 ml. of tris-sucrose buffer (average protein 2·2 mg./ml. of suspension). This final supernatant was retained as the soluble fraction (average protein 10 mg./ml.).

Total lactate dehydrogenase activity was measured in a reaction mixture containing 0·1 ml. of enzyme, 0·1 ml. of reduced NAD solution (containing 0·30 mg.), 2·7 ml. of 0·1 M phosphate buffer, pH 7·4, and 0·1 ml. sodium pyruvate (0·25 mg.), following the technique of Wroblewski and LaDue (1955). Activity of the enzyme was expressed as m$\mu$ moles of lactate transformed/mg. protein/min.

Isozymes of lactic dehydrogenase were separated on 3·5 × 20 cm. mylar strips (P40 B film leader, 0·004 in. thick, DuPont Corp., Wilmington, Del.) supporting an agarose gel. Separations were carried forward for $1\frac{1}{4}$ hours at 240 volts. LDH isozyme activities were demonstrated* as bands developed by the conversion of nitro blue tetrazolium into formazan. The agarose strips were incubated a

* Isozyme assay (color development, as a function of activity) mixture:

| | |
|---|---|
| Potassium phosphate monobasic | 120 mg. |
| Sodium phosphate dibasic | 664 mg. |
| Sodium cyanide | 24 mg. |
| Magnesium chloride | 8 mg. |
| DL-Lactic acid (Sigma Grade V) | 0·5 ml. |
| p-Nitro blue tetrazolium | 24 mg. |
| Water | 39·5 ml. |
| Phenazine methosulfate (1 mg./1 ml.) | 0·3 ml. |
| $\beta$-NAD | 15 mg. |

47° C. for one hour. After incubation the strips were hydrated and then air dried at room temperature.

Separated LDH isoenzyme bands on agar were quantitated by scanning with a recording densitometer. Percentage of activity of each band was calculated as a function of density from the total LDH activity. The method has been described by Wroblewski and LaDue (1955). Its use was convenient here because the intensity of the stained bands is directly proportional to enzyme activity.

### RESULTS AND DISCUSSION

The data presented in Tables I and II and in the bar graph of Fig. 1 illustrate that marked differences existed when LDH activity of tumor tissue was compared with that of lactating breast tissue from normal animals as a function of the subcellular structural elements with which the active proteins are associated.

In the total cell homogenates LDH V and IV are considerably more active in the tumor than in the normal tissue. III of the tumor was less active than LDH-III of normal tissue and II and I, measurable in the homogenate of normal tissue, were absent or below measurable levels in the tumor homogenates. This characteristic picture probably importantly reflects changes in the molecular population of the cytoplasm which, of course, is included within the homogenate.

FIG. 1.—Bar graph indicating distribution of LDH isozymes in lactating breast tissue and in breast tumor tissues.

TABLE I.—*Average LDH Activity and Isozyme Distribution in Homogenates and in Selected Sub-cell Fractions**

| Source | LDH-V | IV | III | II | I | Total activity |
|---|---|---|---|---|---|---|
| Homogenate | | | | | | |
| Breast tissue | 85 | 90 | 54 | 26 | 3 | 258 |
| Tumor | 116 | 95 | 16 | — | – | 227 |
| 20,000 × *g* pellet | | | | | | |
| Breast tissue | 23 | 31 | 4 | — | – | 58 |
| Tumor | 36 | 17 | — | — | – | 53 |
| 100,000 × *g* pellet | | | | | | |
| Breast tissue | 54 | 50 | 19 | 3 | – | 126 |
| Tumor | 93 | 56 | 6 | — | – | 155 |
| 100,000 × *g* supernate | | | | | | |
| Breast tissue | 81 | 86 | 65 | 38 | – | 270 |
| Tumor | 116 | 91 | 21 | — | – | 228 |

* Activity m$\mu$ moles/mg. protein/min.

In the soluble cell-fraction (100,000 × *g*, 0·25 M sucrose, supernatant), in the same manner as in the homogenate, LDH-IV and V were considerably more active in the tumor than in the normal cell preparation. LDH-III was less active in tumors than in normal tissue, and LDH-II, present in the normal, is absent in the tumor tissue. LDH-I was absent or below measurable levels in both normal and tumor tissue.

The 100,000 × *g* pellet, which consisted largely of endoplasmic reticulum, showed a marked elevation of LDH-V but a small, though presumably significant diminution of LDH-IV. LDH-III in this fraction was markedly less active in the tumor than in the homologous normal preparation and LDH-II while low is measurable in the normal 100,000 × *g* fraction but is below measurable levels in the tumor preparation. Lactate dehydrogenase isozyme-I was absent in both normal and in tumor 100,000 × *g* pellet.

The 20,000 × *g* pellet, representing principally fragments of cell nuclei, shows a marked elevation of LDH-V in the tumor when it is compared with the normal. LDH-IV, contrariwise, is less active in tumor tissue than it is in the lactating breast tissue. LDH-III, unmeasurable in the tumor, is present at a moderate level of activity in normal breast. Isozymes II and I are absent or unmeasurable in both normal and tumor nuclear isolations.

An observation which can be made in the analysis of this data is that the several LDH isozymes tend to represent a population distribution which is discrete. Changes measured in total homogenates (and presumably those which might be found in serum) represent average changes that can be resolved by separation of subcellular components. One may find possible portent in this distribution of isozyme activities. In cytoplasm and in total cell homogenates LDH-IV is clearly more active in the tumor than in the normal homologous tissue but in the membrane cell component (endoplasmic reticulum fraction) LDH-IV is less active in the tumor than in the normal tissue. Even more dramatically, the nuclear fraction LDH-IV has a lower activity in the tumor than does the same isozyme in the homologous tissue.

It is evident that an interpretation of lactic dehydrogenase changes in neoplasia must be undertaken, not solely upon measurements which involve serum or even tissue homogenates. Rather, changes of significance in neoplasia may be most

TABLE II.—*Per cent LDH Isozyme Distribution in Subcellular Fractions of Breast Tissue and Tumor Tissue*

| Source | V | IV | III | II | I |
|---|---|---|---|---|---|
| Homogenate | | | | | |
| Breast tissue | 28 | 32 | 30 | 10 | 0 |
| | 30 | 34 | 22 | 12 | 2 |
| | 38 | 40 | 12 | 8 | 2 |
| | 34 | 36 | 20 | 9 | 1 |
| Average | 33 | 35 | 21 | 10 | 1 |
| Tumor | 56 | 41 | 3 | — | – |
| | 50 | 44 | 6 | — | – |
| | 63 | 32 | 5 | — | – |
| | 52 | 35 | 13 | — | – |
| | 47 | 45 | 8 | — | – |
| | 56 | 44 | — | — | – |
| | 39 | 55 | 6 | — | – |
| | 51 | 40 | 9 | — | – |
| Average | 51 | 42 | 7 | — | – |
| 100,000 × *g* supernate | | | | | |
| Breast tissue | 28 | 35 | 25 | 12 | – |
| | 31 | 31 | 23 | 15 | – |
| | 27 | 28 | 28 | 17 | – |
| | 34 | 35 | 20 | 11 | – |
| Average | 30 | 32 | 24 | 14 | – |
| Tumor | 53 | 40 | 7 | — | – |
| | 48 | 44 | 8 | — | – |
| | 52 | 40 | 8 | — | – |
| | 38 | 47 | 15 | — | – |
| | 47 | 41 | 12 | — | – |
| | 64 | 26 | 10 | — | – |
| | 57 | 33 | 10 | — | – |
| | 45 | 46 | 9 | — | – |
| | 51 | 43 | 6 | — | – |
| Average | 51 | 40 | 9 | — | – |
| 20,000 × *g* pellet | | | | | |
| Breast tissue | 40 | 54 | 6 | — | – |
| | 37 | 57 | 6 | — | – |
| | 42 | 50 | 8 | — | – |
| | 42 | 50 | 8 | — | – |
| Average | 40 | 53 | 7 | — | – |
| Tumor | 60 | 40 | — | — | – |
| | 44 | 56 | — | — | – |
| | 71 | 29 | — | — | – |
| | 62 | 38 | — | — | – |
| | 72 | 28 | — | — | – |
| | 80 | 20 | — | — | – |
| | 89 | 11 | — | — | – |
| | 63 | 37 | — | — | – |
| | 74 | 26 | — | — | – |
| Average | 68 | 32 | — | — | – |
| 100,000 × *g* pellet | | | | | |
| Breast tissue | 40 | 43 | 15 | 2 | – |
| | 45 | 38 | 14 | 3 | – |
| | 45 | 38 | 15 | 2 | – |
| | 41 | 43 | 14 | 2 | – |
| Average | 43 | 40 | 15 | 2 | – |
| Tumor | 60 | 37 | 3 | — | – |
| | 62 | 35 | 3 | — | – |
| | 60 | 36 | 4 | — | – |
| | 57 | 43 | — | — | – |
| | 62 | 30 | 8 | — | – |
| | 64 | 36 | — | — | – |
| | 60 | 36 | 4 | — | – |
| | 64 | 36 | — | — | – |
| Average | 61 | 36 | 3 | — | – |

pertinently referred to subcellular fractions. Surely, at the least, no cohesive interpretation of the relationship of lactate dehydrogenase isozymes to changes in malignancy can be undertaken without reference to the discrete molecular populations which have been shown to exist.

SUMMARY

An examination of lactic dehydrogenase activity patterns in neoplasia has been described. Evidence is presented that indicates measurements which involve serum or tissue homogenates could better be replaced by activity measurement within subcellular fractions. Significant, reproducible changes associated with malignancy are shown to be related to discrete molecular population.

This research was supported by USPHS research grant CA08023–04. The assistance of Dr. Yeu-Tsu Lee in the surgical removal of glandular breast tissue is gratefully acknowledged.

### REFERENCES

Damm, H. C., Besch, P. K. and Goldwyn, A. J. (Editors).—(1966) ‘The Handbook of Biochemistry and Biophysics’. Cleveland and New York. (The World Publishing Co.)

Goldman, R. D., Kaplan, N. O. and Hall, T. C.—(1964) *Cancer Res.*, **24**, 389.

Lowry, O. H., Rosebrough, N. J., Farr, A. L. and Randall, R. J.—(1951) *J. biol. Chem.*, **193**, 265.

Markert, C. L. and Möller, F.—(1959) *Proc. natn. Acad. Sci. U.S.A.*, **45**, 759.

Ono, T.—(1966) *In* ‘Biological and Biochemical Evaluation of Malignancy in Experimental Hepatomas’, edited by Tomizo Yoshida, Tokyo (Japanese Cancer Association), p. 189.

Spjut, H. J. and Spratt, J. S.—(1965) *Ann. Surg.*, **161**, 309.

Warburg, O.—(1930) ‘Metabolism of Tumors’. London (Constable Press).—(1956) *Science, N.Y.*, **123**, 309.

Winer, A. D. and Schwert, G. W.—(1957) *Fedn Proc. Fedn Am. Socs exp. Biol.*, **16**, 272.

Wroblewski, F. and Ladue, J. S.—(1955) *Proc. Soc. exp. Biol. Med.*, **90**, 210.